# High Dose Parenteral Ascorbate Inhibited Pancreatic Cancer Growth and Metastasis: Mechanisms and a Phase I/IIa study

**DOI:** 10.1038/s41598-017-17568-8

**Published:** 2017-12-07

**Authors:** Kishore Polireddy, Ruochen Dong, Gregory Reed, Jun Yu, Ping Chen, Stephen Williamson, Pierre-Christian Violet, Ziyan Pessetto, Andrew K. Godwin, Fang Fan, Mark Levine, Jeanne A. Drisko, Qi Chen

**Affiliations:** 1 Department of Pharmacology, Toxicology and Therapeutics, University of Kansas Medical Center, Kansas City, KS 66160 USA; 2 Integrative Medicine, University of Kansas Medical Center, Kansas City, KS 66160 USA; 3 Department of Internal Medicine, Hematology and Oncology Division, University of Kansas Medical Center, Kansas City, KS 66160 USA; 4 Department of Pathology and Laboratory Medicine, University of Kansas Medical Center, Kansas City, KS 66160 USA; 5 National Institute of Diabetes, Digestive and Kidney Diseases, the National Institutes of Health, Bethesda, MD 20892 USA

## Abstract

Pancreatic cancer is among the most lethal cancers with poorly tolerated treatments. There is increasing interest in using high-dose intravenous ascorbate (IVC) in treating this disease partially because of its low toxicity. IVC bypasses bioavailability barriers of oral ingestion, provides pharmacological concentrations in tissues, and exhibits selective cytotoxic effects in cancer cells through peroxide formation. Here, we further revealed its anti-pancreatic cancer mechanisms and conducted a phase I/IIa study to investigate pharmacokinetic interaction between IVC and gemcitabine. Pharmacological ascorbate induced cell death in pancreatic cancer cells with diverse mutational backgrounds. Pharmacological ascorbate depleted cellular NAD+ preferentially in cancer cells versus normal cells, leading to depletion of ATP and robustly increased α-tubulin acetylation in cancer cells. While ATP depletion led to cell death, over-acetylated tubulin led to inhibition of motility and mitosis. Collagen was increased, and cancer cell epithelial-mesenchymal transition (EMT) was inhibited, accompanied with inhibition in metastasis. IVC was safe in patients and showed the possibility to prolong patient survival. There was no interference to gemcitabine pharmacokinetics by IVC administration. Taken together, these data revealed a multi-targeting mechanism of pharmacological ascorbate’s anti-cancer action, with minimal toxicity, and provided guidance to design larger definitive trials testing efficacy of IVC in treating advanced pancreatic cancer.

## Introduction

Whereas advancements in molecular and targeted therapies have greatly improved survival of patients with many types of cancers, treatment outcomes for pancreatic cancer have not changed significantly over the past 30 years. Pancreatic cancer remains the most fatal type of cancer, with 5-year survival less than 8%^1^. If left untreated, the median life expectancy is 3½ months after diagnosis. Gemcitabine monotherapy was the standard of care for more than 15 years^2^. However, it produces a median overall survival (OS) duration of only 6–7 months, with little impact on OS of patients with locally advanced or metastatic disease, who comprise the majority of cases^3^. Recently developed combination regimens such as FOLFIRINOX^4^ or gemcitabine plus nab-paclitaxel^5^ have prolonged the median OS to 8.5–13 months, but with added significant toxic burden. Numerous attempts have been made to improve systemic therapies, but they have either failed to improve efficacy or added significant toxic side effects^6–8^.

Recently, there has been increased interest in using high-dose intravenous ascorbate (IVC) as an adjunct therapy with standard chemotherapy^9^. IVC is safe and free of common toxic side effects that often accompany chemotherapies. A phase I trial by Hoffer *et al*.^10^ treating 24 terminal cancer patients found that IVC dosed at 1.5 g/kg 3x weekly was free of significant toxicity, and unexpectedly, 2 patients had stable disease. Our group reported a pilot trial treating stage III-IV ovarian cancer patients^9^ randomized to the standard paclitaxel/carboplatin chemotherapy, or the standard chemotherapy plus IVC (75–100 g/infusion, 2 x weekly for 1 year). IVC treatment substantially decreased chemo-associated toxicities^9^. The median time for disease progression/relapse was prolonged by 8.75 months in the ascorbate + chemo group compared to the chemo-only group, despite the trial not being statistically powered to detect efficacy^9^. Two small trials in pancreatic cancer patients were recently reported by Monti *et al*.^11^ and Cullen *et al*.^12^ both using IVC (50–100 g/infusion 2–3x weekly) together with gemcitabine or gemcitabine plus the EGFR inhibitor, erlotinib. In both trials, IVC did not increase any toxicity to the chemotherapy. In Monti’s trial, 8 out of 9 patients had tumor shrinkage after only 8 weeks of treatment^11^. In Cullen’s trial, despite its very small size, overall survival was nearly doubled compared to historical controls^12^. Similar good tolerability was reported in non-small-cell lung cancer (NSCLC) and glioblastoma multiforme (GBM) patients^13^. A recent mechanistic study showed that ascorbate had preferential cytotoxic effects against KRAS and BRAF mutated colon cancer cells^14^. Since more than 90% of pancreatic cancers harbor KRAS mutations^15^, there is great potential to use IVC as a low-toxic treatment for pancreatic cancer.

In contrast to oral ingestion of vitamin C, IVC bypasses the physiological “tight control” of systemic concentrations and achieves pharmacological concentrations^16–18^. Pharmacological concentrations of ascorbate (Asc) generate hydrogen peroxide, which via Haber-Weiss reaction and Fenton chemistry induces oxidative damage^9,19–21^. The ascorbate-induced cytotoxicity apparently is selective to different types of cells, with higher toxicity toward cancer cells relative to normal cells^22^. Many laboratories have shown in rodent xenografts that Asc decreased the growth rate of various aggressive tumors without causing adverse effects, such as pancreatic cancer, glioblastoma, ovarian cancer, prostate cancer, hepatoma, colon cancer, sarcoma, leukemia, mesothelioma, breast cancer, and neuroblastoma^19–21,23–27^. Our previous study, using a panel of 7 pancreatic cancer cell lines, showed that Asc sensitized all these pancreatic cancer cells to gemcitabine treatment, despite their different genetic backgrounds^28^. In this study we investigated the mechanisms of Asc in inhibiting pancreatic cancer growth and metastasis, evaluated the safety when adding IVC to gemcitabine chemotherapy, and assessed pharmacokinetics to determine whether there is drug-drug interaction when administering IVC and gemcitabine concurrently.

## Results

### Mechanisms of pharmacological ascorbate decreasing pancreatic cancer cell viability and metastasis

**Figure 1:**
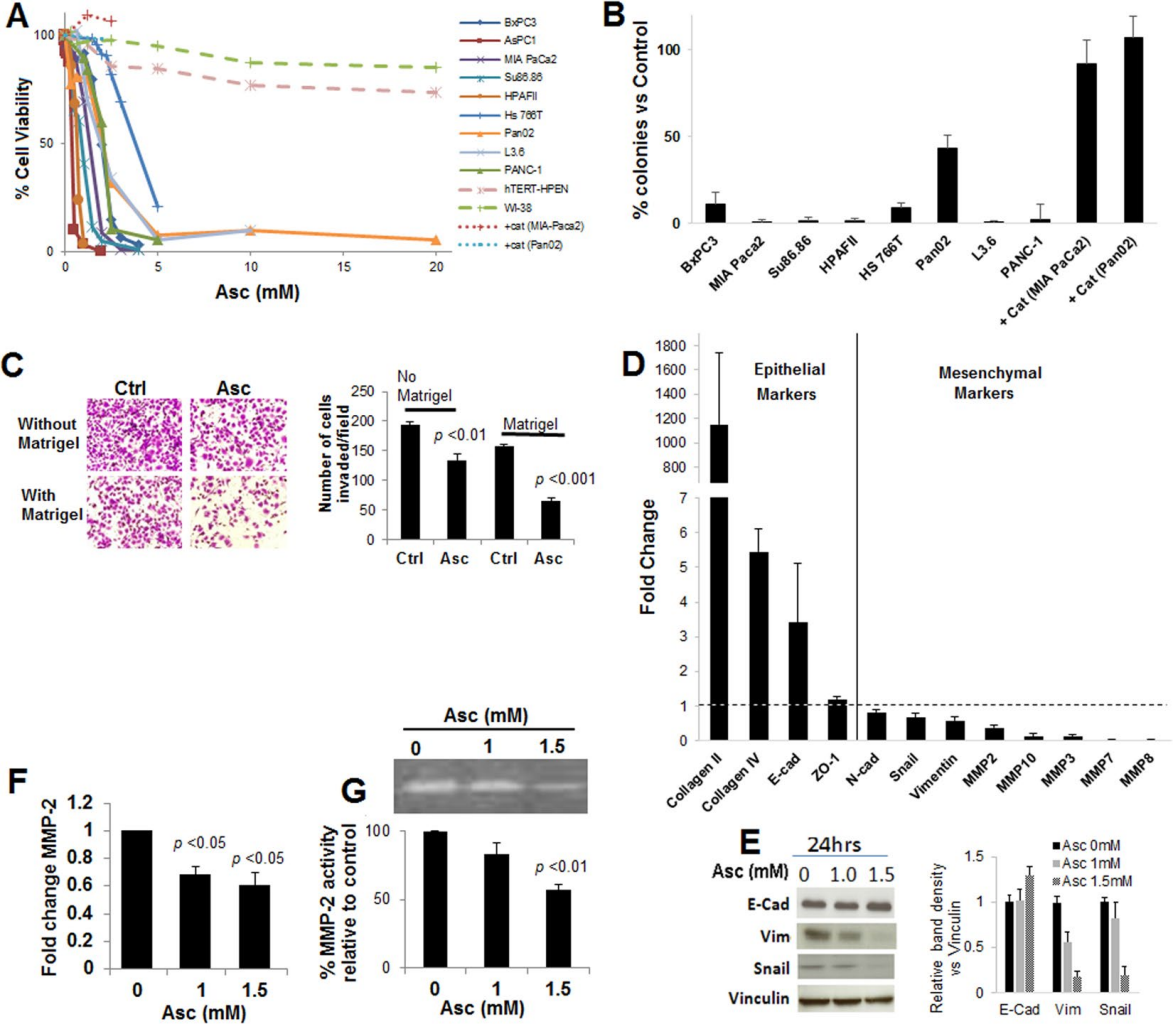
Ascorbate inhibited pancreatic cancer growth and metastasis *in vitro*. (**A**) Dose responses of pancreatic cancer cells and normal cells to ascorbate. Cells were exposed to 0–20 mM ascorbate and cell viability was detected at 24 h by MTT assays. +cat, pre-incubation with 600 U/ml catalase. Data represents Mean ± SD of 3 experiments each done in triplicates. (**B**) Colony formation of pancreatic cancer cells under ascorbate treatment. Cells were exposed to 5 mM of ascorbate and colonies were counted at 21–28 days. Data shows % of colonies relative to untreated control (Mean ± SD of ≥3 replicates). (**C**) Matrigel invasion assays for pancreatic cancer cell migration and invasion. PANC-1 cells were seeded at 1 × 10^4^ cells/insert and were exposed to 1 mM ascorbate (Asc). Cell migration (without Matrigel) and invasion (with Matrigel) were detected at 24 hrs. Bar graph shows the average number of migrated/invaded cells per field. Data represents ≥ 3 experiments each done in triplicates. **(D)** qRT-PCR for changes in EMT markers. PANC-1 cells were treated with 1.5 mM Asc for 24 hrs. Data was normalized to 18s rRNA, and then compared to control PANC-1 cells for fold change. Data represent Mean ± SD of 2–6 independent experiments. (**E**) Western blot in PANC-1 cells showing expression of E-cadherin (E-Cad), vimentin (Vim) and Snail after ascorbate treatment. Vinculin was a loading control. Left panel shows representative blots. Bar graph shows relative band density normalized to vinculin, analyzed by Image J. (**F**) qRT-PCR for MMP-2 mRNA expression in PANC-1 cells treated with ascorbate for 24 hrs. Data represents Mean ± SD of 3 independent experiments. (**G**) Gelatin zymography assay for MMP-2 enzymatic activity after ascorbate treatment. After ascorbate treatment cell lysate were used for RNA isolation, reverse transcription and qRT-PCR. Supernatant media was used for detection of MMP-2 activity using gelatin zymography. Data represents Mean ± SD of 3 independent experiments.

A panel of 8 human pancreatic cancer cell lines and 1 murine pancreatic cancer cell line were exposed to up to 20 mM of ascorbate, which are clinically relevant concentrations^19^. These cells harbor different combinations of genetic alternations that are the most commonly seen in pancreatic cancer patients, including different mutational status of KRAS and P53 (Table S1). Viability in all tested cancer cells was markedly decreased 24 to 48 h after the treatment, despite their different genetic backgrounds. For all tested pancreatic cancer cell lines, IC50 values were below 5 mM (Fig. 1A). In contrast, the treatment of 20 mM ascorbate only minimally influenced viability of a non-tumorigenic pancreatic ductal epithelial cell hTERT-HPEN and fibroblasts (WI-38). Addition of catalase, an enzyme that specifically degrades hydrogen peroxide (H_2_O_2_), completely reversed ascorbate-induced cell death in cancer cells (Fig. 1A). To assess long-term inhibition in cancer cells, colony formation ability of the cells in soft agar was evaluated. Ascorbate at 5 mM significantly decreased the percentage of colony formation in all tested cancer cells (Fig. 1B). Again, catalase completely reversed the inhibitory effect of pharmacological ascorbate. These data confirmed conclusions from previous studies that high concentrations of ascorbate selectively induced cell death in cancer cells versus normal cells, through H_2_O_2_ formation^20,21^. Given the promiscuity of H_2_O_2_ as a prodrug for reactive oxygen species (ROS), and the sensitivity of cells to ascorbate despite a variety of mutations, we postulated that pharmacological ascorbate would target multiple pathways in a cancer cell. As clinical data suggest that high dose intravenous ascorbate has minimal toxicity^9–12^, targeting multiple pathways for cancer treatment is advantageous because of potential synergy.

Interestingly, at a sub-cytotoxic concentration of 1 mM, ascorbate was able to inhibit PANC-1 cell migration and invasion through Matrigel coated Boyden chambers (Fig. 1C), without influencing viability of the cells (Fig. S1). Because cancer cell epithelial-mesenchymal transition (EMT) has been suggested to be the initial step of cancer cell dissemination and metastasis^29^, we first examined whether ascorbate treatment influenced the EMT features of pancreatic cancer cells. In PANC-1 cells treated with sub-cytotoxic concentrations of Asc (1–1.5 mM), E-cadherin expression was increased as shown by both RT-PCR and western blot (Fig. 1D,E). Consistently, Snail, a major EMT transcription factor, was decreased, and the mesenchymal markers Vimentin and N-cadherin were also decreased (Fig. 1D,E). The overall pattern in the changes of EMT markers indicated attenuation of EMT. Similar results were seen in another pancreatic cancer cell line MIA PaCa2 (Fig. S2). Remarkably, mRNAs of collagens were dramatically increased in Asc treated PANC-1 cells (Fig. 1D). At the same time, mRNAs of multiple matrix metalloproteinases (MMPs) were decreased (Fig. 1D). We further examined MMP-2 as a representative^30^. Asc treatment decreased MMP-2 expression dose dependently in PANC-1 cells in 24 h of treatment (Fig. 1F). Gelatinolytic activity of secreted MMP-2 also decreased dose dependently to Asc in the supernatant media of PANC-1 cells (Fig. 1G). These data indicated that even at sub-cytotoxic concentrations, ascorbate exerted actions in inhibiting pancreatic cancer cell migration and invasion.

**Figure 2:**
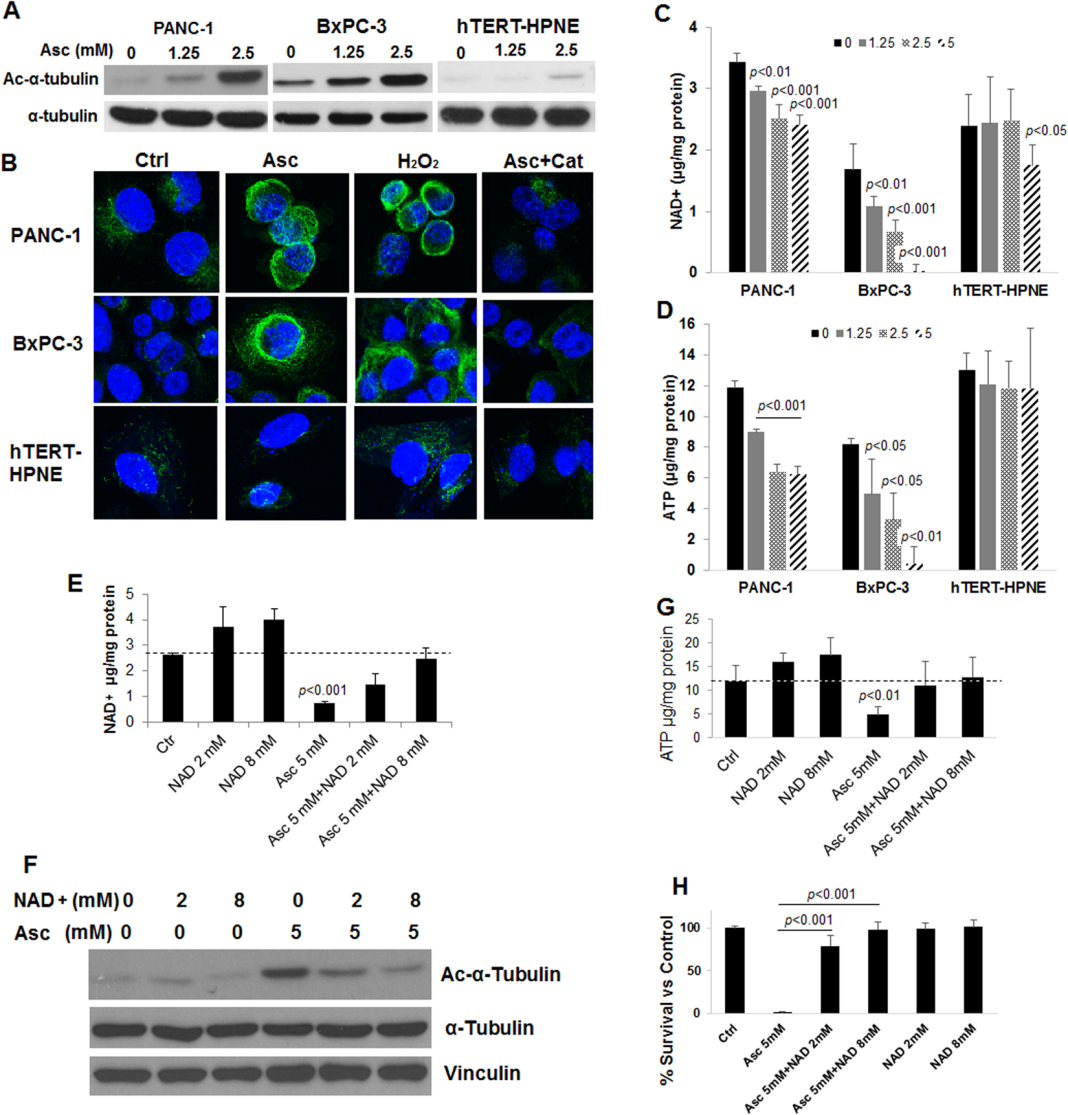
Ascorbate depleted NAD+ in pancreatic cancer cells and enhanced tubulin acetylation. (**A**) Western blot analysis of acetylated α-tubulin in pancreatic cancer cells (BxPC-3 and PANC-1), and an immortalized non-cancerous pancreatic ductal epithelial cell line hTERT-HPNE. Cells were treated for 4 hrs. (**B**) Immunofluorescence with confocal microscopy showing acetylated α-tubulin in cells treated with ascorbate (Asc) 2.5 mM or hydrogen peroxide (H_2_O_2_) 500 µM for 4 hrs. Asc + Cat, co-treatment of ascorbate and 600 U/ml catalase for 4 hrs. Cell nuclei were counter-stained blue with hoechst33342 (2 mg/mL). (**C**) Changes of NAD+ and (**D**) ATP in PANC-1, BxPC-3 and hTERT-HPNE cells treated with Asc for 4 hrs. NAD+ and ATP were detected by HPLC-UV analysis and normalized to protein contents. Data represents Mean ± SD of 2–4 independent experiments. (**E**) Supplementation of NAD+ protected PANC-1 cellular NAD+ levels with Asc treatment. (**F**) Western blot showing tubulin acetylation was prevented with supplementation of NAD+. (**G**) ATP in PANC-1 was protected with supplementation of NAD+. (**H**) Colony formation was protected with supplementation of NAD+. PANC-1 cells were pre-incubated with NAD+ for 30 min, and then treated with Asc and seeded into 2-layer soft agar for colony formation. Colonies were counted after 21 days of incubation. NAD+, tubulin acetylation, and ATP were detected at 4 h of treatment. Data represents Mean ± SD of ≥3 independent experiments.

Because pharmacological ascorbate was synergistic with paclitaxel^9^, and paclitaxel stabilizes microtubules, we investigated whether pharmacological ascorbate could act similarly, by acetylating tubulin and thereby interfering with its catabolism specifically in cancer cells. Robust α-tubulin acetylation was found in PANC-1 and BxPC-3 cells treated with Asc at both sub-cytotoxic and cytotoxic concentrations (1.25–2.5 mM), in a dose dependent manner (Fig. 2A,B). In contrast, Asc only minimally increased acetylated α-tubulin in the non-cancerous pancreatic ductal epithelial cell hTERT-HPEN. H_2_O_2_ treatment mimicked the effects of Asc, whereas catalase completely eradicated α-tubulin acetylation induced by Asc (Fig. 2B). These data indicate that the mechanism of Asc action in stabilizing α-tubulin is dependent on extracellular H_2_O_2_ generated by pharmacological ascorbate^21^. Previous studies reported DNA damage and subsequent PARP activation in cancer cells treated with Asc^9^. As activated PARP utilizes NAD+ as substrate^20,31^, we hypothesized that NAD+ is decreased by Asc treatment. Indeed, NAD+ levels significantly dropped in PANC-1 and BxPC-3 cells in relation to Asc concentrations, within 4 hours of exposure when cell death had not occurred (Fig. 2C). The decrease of NAD+ caused depletion in ATP in PANC-1 and BxPC-3 cells (Fig. 2D), which is related to the subsequent cell death and inhibition in proliferation. In the non-cancerous hTERT-HPEN cells, the 1.25–2.5 mM ascorbate concentrations had no effects on the level of NAD+, only the 5 mM concentration caused decrease in NAD+, and the decrease was more subtle compared to that in cancer cells (Fig. 2C). There was no decrease in cellular ATP levels in hTERT-HPEN cells (Fig. 2D). The selectivity of Asc effects could be explained by two reasons: 1) active replication renders cancer cell DNA more susceptible to oxidative stress^32^ and thus NAD+ is under a greater demand by PARP in cancer cells; 2) the Warburg effect^9,33^ showed that cancer cells depend more on glycolysis for ATP production, which is inefficient compared to normal oxidative phosphorylation pathway. Therefore, depletion of NAD+ induced catastrophic effects on cancer cells’ ATP production while sparing normal cells.

In addition, because NAD+ is an essential co-factor for the tubulin deacetylzing enzyme Sirt-2^34^, the decrease of NAD+ inhibited the activity of Sirt-2 and resulted in increased α-tubulin acetylation, even when there was no change in Sirt-2 protein levels with Asc treatment (Fig. S2A). As evidence, in PANC-1 cells, supplementation of NAD+ to the cell culture media rescued the NAD+ decrease caused by a cytotoxic concentration of 5 mM Asc (Fig. 2E) thus reversing the Asc-mediated α-tubulin acetylation in a dose dependent manner (Fig. 2F). Cellular ATP was also rescued (Fig. 2G). As ATP was protected and the excess tubulin acetylation was prevented, cell viability as measured by colony formation was restored to control values (Fig. 2H).

Because α-tubulin acetylation is under the balanced control of acetyl transferases (α-TAT) and deacetylases (Sirt-2 and HDAC6)^35^, we also examined α-TAT and HDAC6. No change was found in α-TAT with ascorbate treatment, while decrease in HDAC6 expression was found in both PANC-1 and BxPC-3 cells treated with Asc (Fig. S2A). However, overexpression of HDAC6 in PANC-1 cells only slightly reversed α-tubulin acetylation (Fig. S2B), and did not influence Asc-induced cell death (Fig. S2C). Although data here cannot exclude the contribution of HDAC6 and α-TAT, a major role of Sirt-2 is likely because supplementation of NAD+ completely rescued Asc-induced α-tubulin acetylation and cell death.

**Figure 3:**
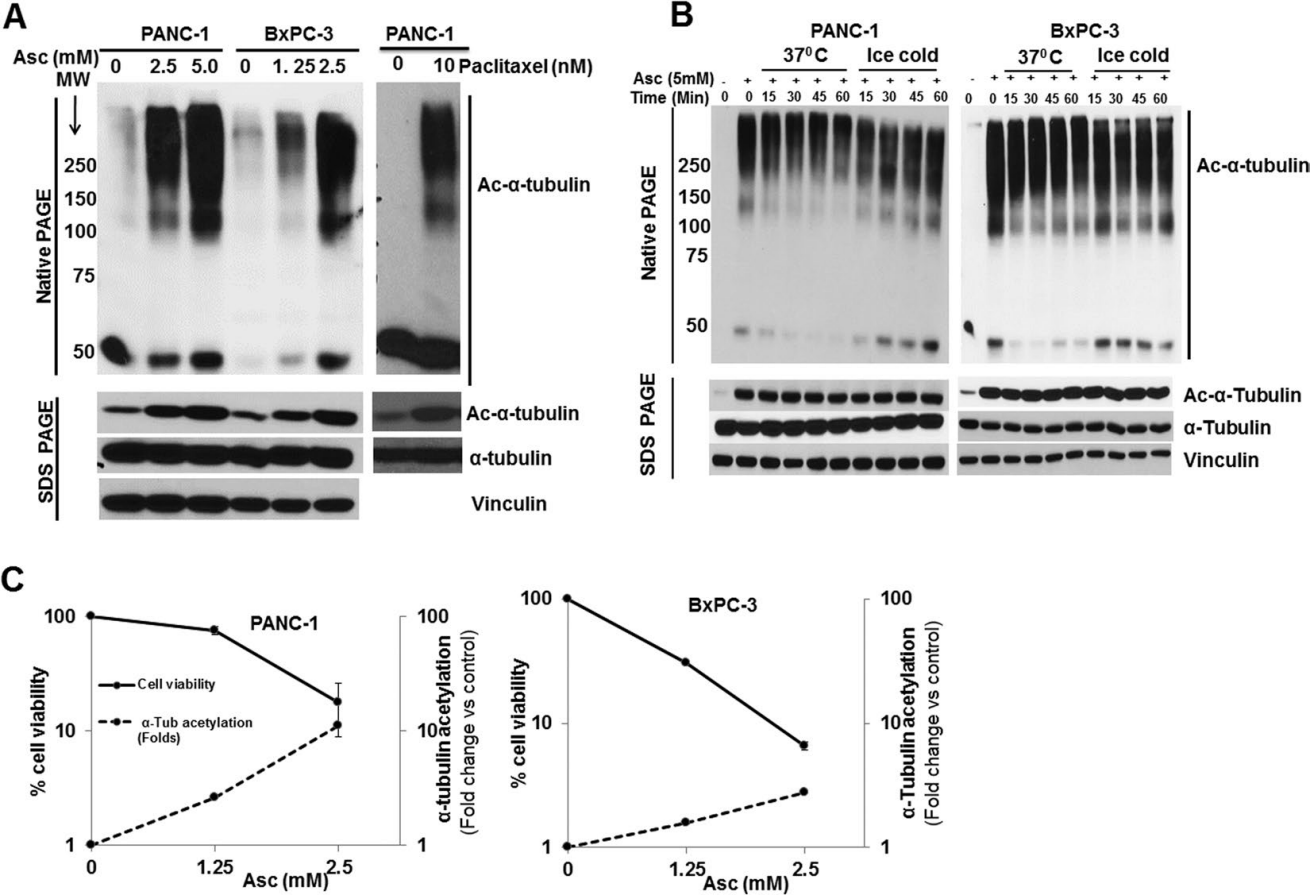
Ascorbate over-stabilized polymerized tubulin by increased tubulin acetylation. (**A**) Tubulin Polymerization detected by native PAGE in PANC-1 and BxPC-3 cells. After 4 h of Asc or paclitaxel treatment, cells were lysed in RIPA buffer and subject to native PAGE. α-tubulin acetylation was confirmed by SDS PAGE and western blot as shown in the bottom panels. MW, molecular weight in kDa. (**B**) Cold induced microtubule depolymerization assay in PANC-1 and BxPC-3 pancreatic cancer cells. After Asc treatment for 4 h cell lysates were exposed to either 37 °C or sit on ice (4 °C) for indicated time, and were then subject to native PAGE. (**C**) Correlation between tubulin acetylation and cell death induced by ascorbate. All results are representatives of ≥3 independent experiments.

Acetylated α-tubulin is associated with stable microtubules^36^. Here, we found over-stabilization of microtubules induced by Asc treatment. Four hours after Asc treatment, there was an enrichment of high molecular weight fractions of acetylated α-tubulin, indicating microtubule polymerization, mimicking the effect of paclitaxel (Fig. 3A). As cold temperature is known to induce depolymerization of tubulin^37^, cells lysates were put on ice (4 °C), and the Asc-induced tubulin polymerization was found stable over time (Fig. 3B). The degree of α-tubulin acetylation was inversely correlated to the viability of pancreatic cancer cells after Asc treatment (PANC-1, r = −0.98287 and BxPC-3, r = −0.88609, by Pears test) (Fig. 3C).

### Inhibition of pancreatic cancer growth and metastasis by pharmacological ascorbate in a mouse model

**Figure 4:**
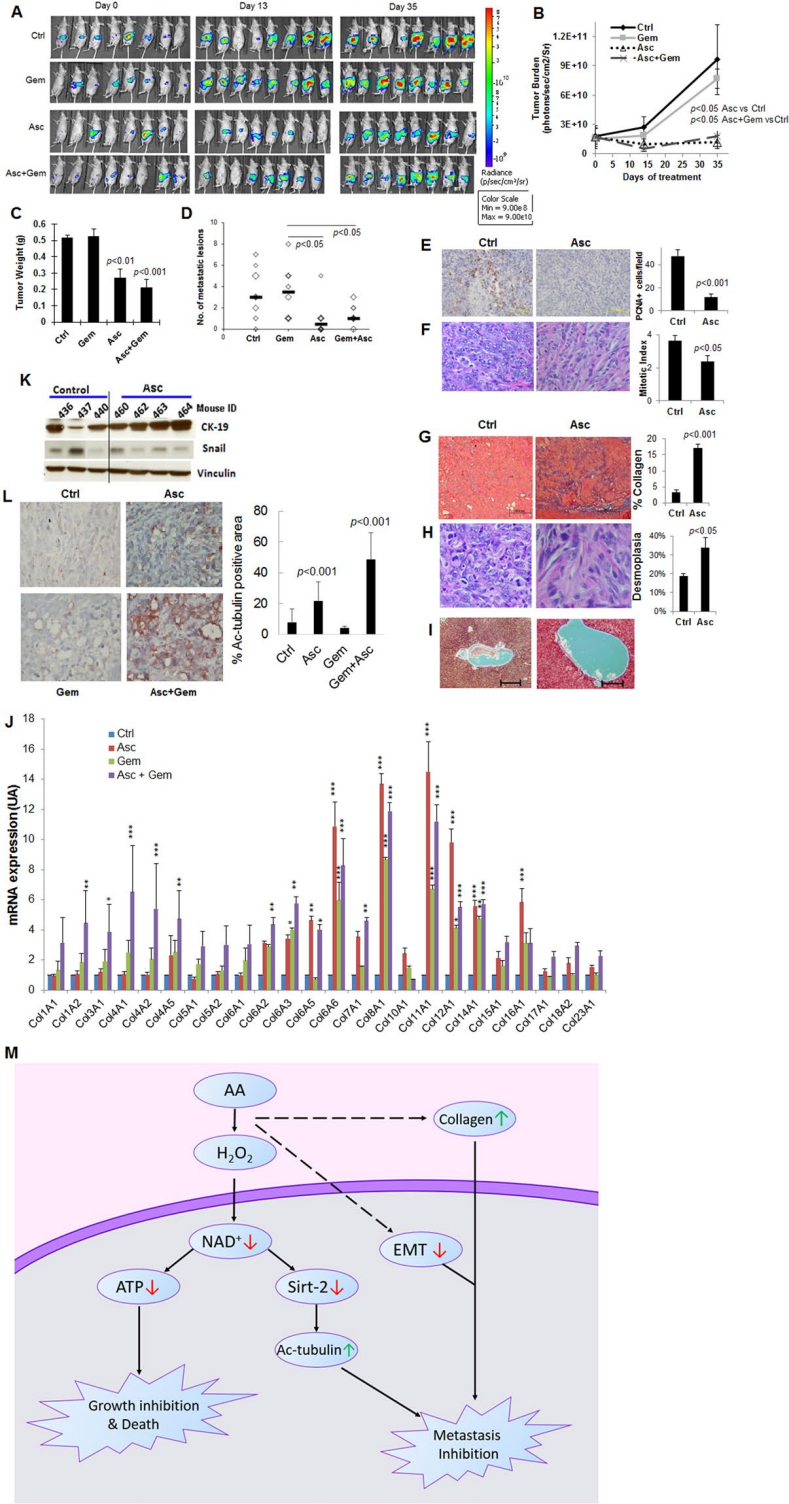
Ascorbate inhibited pancreatic cancer growth and metastasis *in vivo*. (**A**) Bioluminescence images of mice bearing orthotopic pancreatic xenografts treated with ascorbate (Asc), gemcitabine (Gem) or the combination of Asc+Gem. Day 0 indicated the beginning of treatment which was 2 weeks post orthotopic injection of luciferase expressing PANC-1-Leu cells into mouse pancreas. Day 45 was the end of the experiment. Asc, ascorbate treatment at intraperitoneal dose of 4 g/kg/day. Gem, gemcitabine at intraperitoneal dose of 40 mg/kg/week. Control (Ctrl) mice were treated with saline that had the same osmolarity as the ascorbate injections. (**B**) Total tumor burden per mouse by imaging was quantified as photons/sec/cm^2^ (Mean ± SEM). (**C**) Total tumor weight (Mean ± SEM), and (**D**) number of metastatic lesions in each mouse, determined by necropsy at Day 45. (**E**) Immunohistochemical analysis of proliferating cell nuclear antigen (PCNA) with formalin fixed tumor samples. Bar graph (right) represents the average number of PCNA positive cells per field. 15 fields from 3 different tumors from each group were analyzed. (**F**) Histological analysis of mitosis on H&E stained tumor slices. Bar graph (right) shows mitotic index, which was the average number of mitoses from 4 separate fields. Tumors from 4 mice in each group were examined. (**G**) Masson’s trichrome staining for collagen content in tumor tissues. Collagen was stained blue, and cytoplasm pink. Bar graph represents Mean ± SD of % area collagen/cross section. 15 fields from 3 different tumors from each group were analyzed. (**H**) H&E staining for analysis of desmoplasia in mouse tumor tissues. Bar graph shows desmoplasia represented as % of area contains desmoplastic response. Tumors from 4 mice in each group were examined (Mean ± SD). (**I**) Masson’s trichrome staining for collagen and fibrosis of livers from control and ascorbate treated mice. No collagen or fibrosis was seen. (**J**) qRT-PCR detection of 24 kinds of collagen transcripts in mouse tumor samples. Five tumors from each group were detected in duplicates. Bar shows fold changes compared to control mice in Mean ± SD. *P < 0.05; **P < 0.01; and ***P < 0.001. (**K**) Western blot in mouse tumor samples showing changes in CK-19 and Snail. Vinculin was used as loading control. (**L**) Immunohistochemistry in mouse tumor samples showing α-tubulin acetylation. Bar graph represents Mean ± SD of % area of positive staining for acetylated α-tubulin per cross section. 11–17 fields from 3 different tumors from each group were analyzed. (**M**) A simplified scheme showing mechanisms of ascorbate inhibiting pancreatic cancer growth and metastasis.

The multiple mechanisms of Asc actions in pancreatic cancer cells suggested that, inhibition of tumor growth and metastasis was likely to occur *in vivo*. We explored this possibility by using a mouse orthotopic pancreatic cancer model. Luciferase expressing PANC-1 cells were orthotopically injected into pancreas of nude mice. After tumor formation was detected by imaging, mice were grouped and treated with intraperitoneal (IP) doses of ascorbate (Asc) at 4 g /Kg body weight/day for 45 days (equivalent to 1.3 g/Kg/day by intravenous injection)^20^, or gemcitabine (Gem, 40 mg/kg every 3 days, IP), or the combination of Asc and Gem. Live animal imaging showed that the gemcitabine treatment did not inhibit tumor growth. In contrast, Asc treatment alone significantly reduced tumor progress longitudinally (Fig. 4A,B). Combination of gemcitabine and ascorbate (Gem+Asc) achieved significant tumor growth inhibition compared to control and Gem alone groups, but was not different compared to Asc alone group. At the end of the experiment, mice were euthanized and total tumor weight was detected, and visible metastases in the abdomen were examined by gross necropsy. A significant decrease in average tumor weight was found with Asc treated and Gem+Asc treated groups, compared to controls (Fig. 4C). The number of metastases in each mouse were significantly decreased in the Asc group and the Gem+Asc group, compared to controls (Fig. 4D). However, the Gem alone treatment had no effects on both tumor weight and metastases, and the Gem+Asc treatment did not show superior effects compared to Asc treatment alone.

Immunohistochemical analysis on tumor samples showed that proliferating cell nuclear antigen (PCNA), an indicator of cell proliferation, decreased substantially in tumors treated with Asc (Fig. 4E). The mitotic index also decreased significantly in the Asc treated group, as analyzed by hematoxylin and eosin stain (H&E stain) (Fig. 4F). Massive increases in collagen content were found in the tumor stroma of Asc-treated mice (Fig. 4G). RT-PCR detected significant elevation of multiple collagen synthesis in mice tumors with Asc treatment (7 collagen gene transcripts were upregulated in Asc group, 6 in Gem group, and 14 in Gem+Asc group) (Fig. 4J). Considering all transcripts, collagen gene expression in Asc group and Gem+Asc group had significant increase relative to control group (P = 0.001 and 0.00003 respectively); gemcitabine alone did not increase collagen gene expression (P = 0.08); and Gem+Asc group had significant increase relative to Gem group (P = 0.05). Accordingly, desmoplasia in tumor stroma was significantly enhanced in Asc-treated mice (Fig. 4H). No such increase in collagen or fibrosis was found in the livers of the Asc-treated mice (Fig. 4I). Also consistent with cellular data, decrease of Snail was found in mouse tumors treated with ascorbate (Fig. 4K), and the epithelial molecule CK-19 showed robust increases in tumor samples from Asc-treated mice (Fig. 4K). Acetylation of α-tubulin was significantly increased in tumors of Asc-treated mice and in mice treated with Gem+Asc, while Gem treatment alone did not influence α-tubulin acetylation (Fig. 4L).

Taken together, these *in vitro* and *in vivo* data indicated that, pharmacological ascorbate inhibited pancreatic cancer growth and metastasis through a pro-oxidative mechanism, subsequently induced NAD+/ATP depletion selectively in cancer cells, and resulted in inhibition of cell proliferation and induction of cell death. NAD+depletion also caused impairment in Sirt-2 activity, resulted in imbalance of α-tubulin acetylation and subsequently inhibited mitosis and metastasis. Pharmacological ascorbate treatment altered tumor stroma by enhancing collagen production. EMT was inhibited, with mechanisms worthy of further investigation. These multiple mechanisms of pharmacological ascorbate work together and result in inhibition of tumor growth and metastasis in pancreatic tumor (Fig. 4M).

### Safety and tumor response of IVC in pancreatic cancer patients

We conducted a Phase I/IIa trial to investigate safety and pharmacokinetic interaction in pancreatic cancer patients using IVC in combination with gemcitabine. Seven participants were enrolled initially and when safety was confirmed, an additional 7 participants were enrolled (Table S2a,b). Twelve of the 14 enrolled subjects completed phase I pharmacokinetic evaluation composed of IVC and gemcitabine pharmacokinetics each as single drugs followed by pharmacokinetic measurement of IVC combined with gemcitabine (Table S3). These 12 patients entered Phase IIa and received intravenous ascorbate (IVC) 3× weekly at the established doses and in conjunction with gemcitabine on the dose and schedule established by the treating oncologist (Table S4). The treatment continued until tumor progression or patient withdrawal for other reasons (Table S2b).

**Figure 5:**
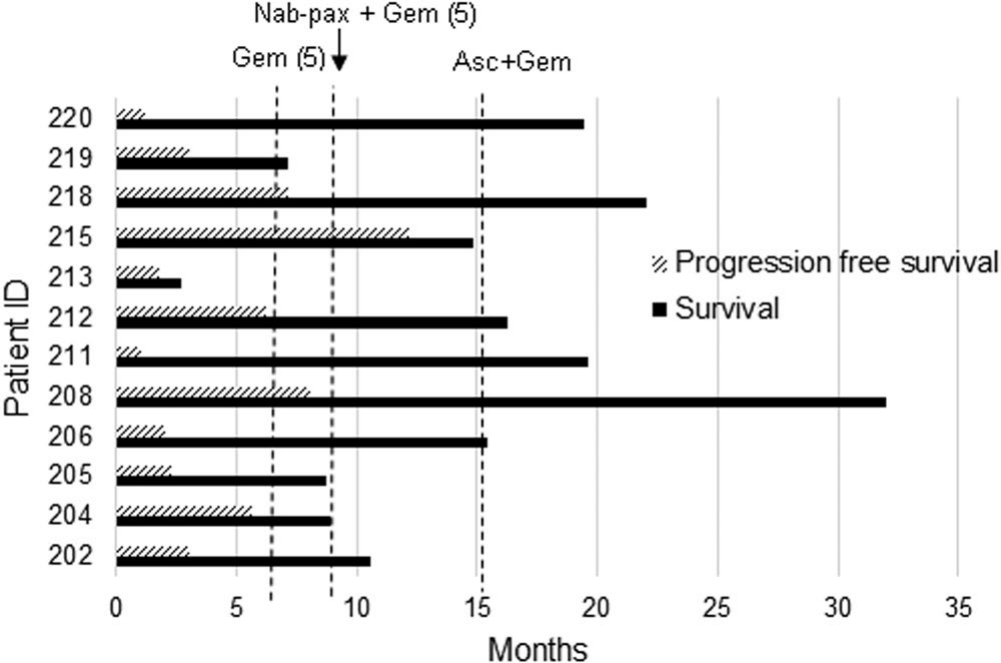
Overall survival (OS) and progression free survival (PFS) of the 12 participants who completed phase IIa study. Dotted lines showed median overall survival.

Of the 12 participants completed Phase IIa treatment, 50% (6/12) survived over 1 year, and 8.3% (1/12) survived more than 2 years after diagnosis. The median overall survival (OS) was 15.1 months (Fig. 5A). Six patients had disease progression based on RECIST criteria and were removed from the study (of these six: 5 had treatment prior to enrollment, 1 without pretreatment); 1 voluntarily withdrew for personal reasons; and 4 were withdrawn based on medical issues not related to disease progression, and 1 withdrew because the treatment response made the participant eligible for surgery. Median progression-free survival (PFS) was 3 months.

One participant (#8) had remarkable tumor response to the treatment regimen. Prior to enrollment, the participant had Stage III pancreatic ductal carcinoma, failed FOLFIRINOX treatment and was on disease progression. The patient was not eligible for surgery because of concerns that the tumor mass involved a mesenteric pancreatic artery (Fig. S3A). After enrollment in the trial, the participant received a total of 70 doses of IVC (64 doses of 100 grams/infusion when in Phase II, and 6 doses from 25–75 g/infusion when in Phase I) and gemcitabine (715 mg/m^2^) for 9 cycles. Imaging showed tumor stabilization/shrinkage and improvement in appearance of margins which became more distinct (Fig. S3A). The patient was then determined suitable for surgical resection. The resected tumor was examined by pathological analysis. Consistent with our pre-clinical data, high levels of collagen content were found in tumor stroma compared to pancreatic ductal carcinoma samples from untreated, FOLFIRINOX treated, or gemcitabine treated patients (Fig. S3B).

Significant adverse events of grade 3 or above (SAE) are shown in Table 1 and none were deemed secondary to the investigational therapy. Multiple Grade 1 and 2 adverse events were found to be usual in the course of conventional therapy and resolved without progression to Grade 3 or above. Adverse events attributable to IVC were Grade 1 nausea and thirst. No other adverse events were found to be related to IVC.

**Table 1:** Significant adverse events (SAEs) at Grade 3 or above for all 14 participants.

ID	Significant Adverse Events	Preexisting Condition	Resolution	DSMB review
1	Grade 3 Gastrointestinal: biliary stent malfunction necessitating hospitalization	Yes	Yes	Not related to study drug
2	No SAE			
3	No SAE			
4	No SAE			
5	No SAE			
6	No SAE			
7	Grade 3 Cardiovascular: with chest pressure, tachycardia, dyspnea, and left arm tingling	Yes	Yes	Not related to study drug
7	Grade 3 Pulmonary: dyspnea	Yes	Yes	Not related to study drug
8	Grade 3 Urinary System: Sepsis gram negative bacteria from UTI	Yes	Yes	Not related to study drug
9	Grade 3 Musculoskeletal: hip fracture from fall with subsequent surgical repair and rehab	No	Yes	Not related to study drug
10	Grade 3 Gastrointestinal: biliary stent malfunction necessitating hospitalization	Yes	Yes	Not related to study drug
10	Grade 3 Cardiovascular: Third degree heart block	Yes	Yes	Not related to study drug
10	Grade 5 Cardiovascular: Myocardial infarction with severe pulmonary edema	Yes	No	Not related to study drug
11	Grade 5 Gastrointestinal: acute onset severe abdominal pain at home with escalating doses of opioids administered; became obtunded and could not be resuscitated by EMT	Yes	No	Not related to study drug
12	No SAE			
13	Grade 3 Neurologic: Cerebrovascular event	Yes	No	Not related to study drug
14	No SAE			

### Effect of IVC on Gemcitabine Pharmacokinetics

**Figure 6:**
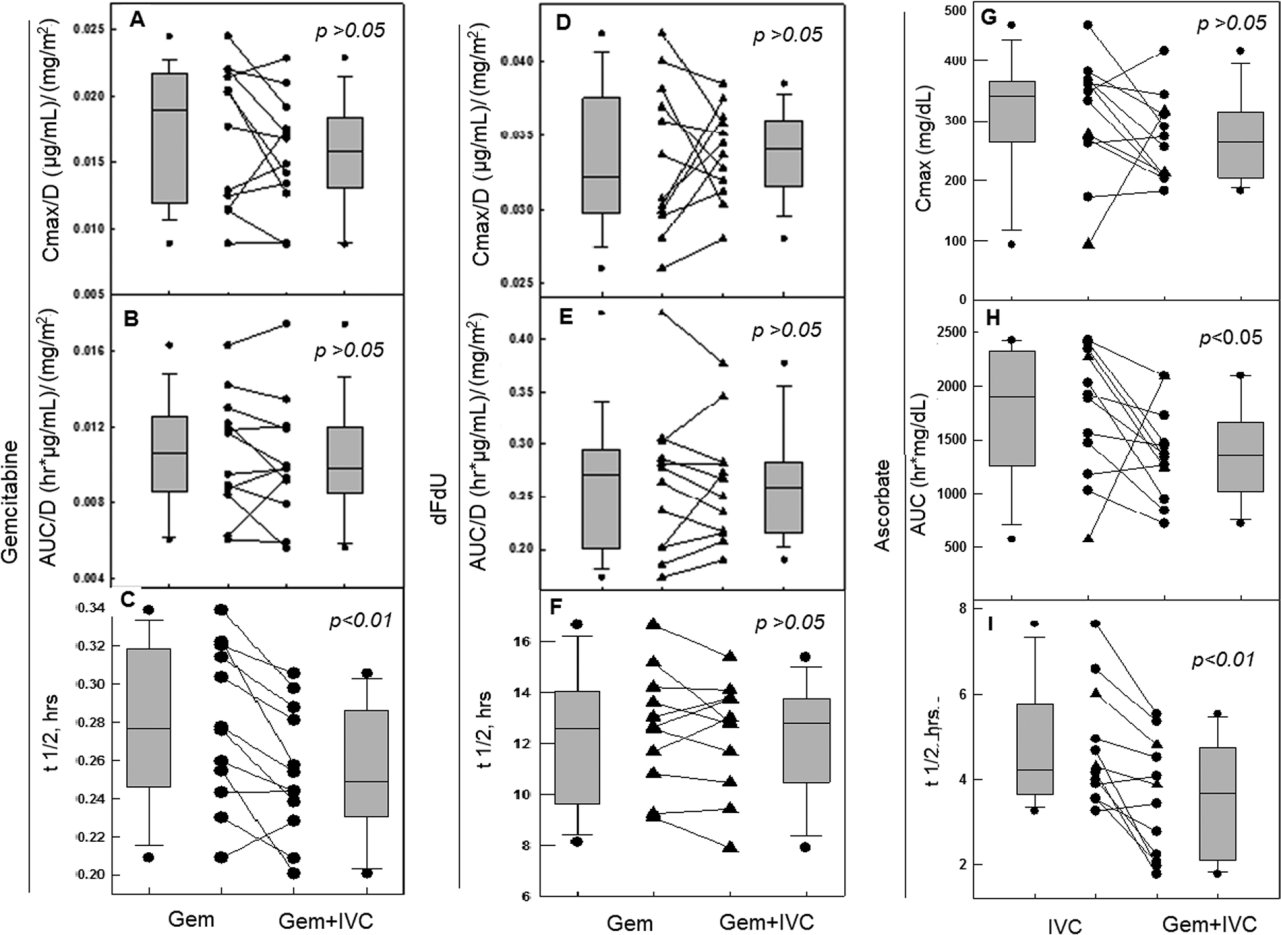
Pharmacokinetic parameters of gemcitabine, dFdU and ascorbate when IVC and gemcitabine were used alone or in combination. Cmax, AUC and t ½ of gemcitabine (**A–C**), dFdU (**D–F**) and ascorbate (**G–I**) were shown. Cmax and AUC were normalized to dose. Line graphs show parameters obtained for each subject from single drug administration (left) and combination administration (right). Box plots denote the median and the 5th, 25th, 75th, and 95th percentiles for all patients, and the outlying values.

Pharmacokinetic parameters of individual participant for gemcitabine, its main metabolite dFdU, and ascorbate were evaluated for the 12 participants, by administering each of IVC and gemcitabine alone, and then in combination (Tables S3, S4). Gemcitabine pharmacokinetics were as expected, with a half-life (t ½) of 15–20 minutes, reflecting rapid deamination to its primary metabolite dFdU, which has a longer t ½ of ~12 hrs. Cmax and AUC of gemcitabine were also comparable to literature reports^38^ (Fig. 6A–C). There was no difference for Cmax and AUC between the values obtained when gemcitabine was administered by itself (Gem) and when it followed IVC (Gem + IVC), either un-normalized (Table S5), or normalized to gemcitabine dose (i.e. Cmax/D and AUC/D) (Fig. 6A,B). Only T ½ of gemcitabine was shortened by 9% (P = 0.003) when combined with IVC (Fig. 6C). As gemcitabine is rapidly metabolized to dFdU, this decrease in t ½ (from 0.28 h to 0.25 h) is unlikely to be clinically significant, and the pharmacokinetics of dFdU should be considered. For dFdU, the values for t ½, Cmax, and AUC were all comparable to previously reported pharmacokinetic parameters^38^, and showed no difference when gemcitabine was administered with ascorbate (Fig. 6D–F).

We further determined the pharmacokinetics of IVC when used alone or in combination with gemcitabine (Fig. 6G–I). Cmax was not changed. Both t ½ and AUC, however, were impacted by gemcitabine. T ½ decreased by 25% and AUC decreased by 20% for ascorbate when it was used with gemcitabine.

Overall, these data clearly indicated that no significant alteration in gemcitabine disposition had occurred related to the use of IVC, however, a small increase in clearance of ascorbate was seen when co-administered with gemcitabine.

## Discussion

Previous studies indicate that hydrogen peroxide (H_2_O_2_) formation drives the actions of pharmacological ascorbate against cancer cells^19–21^. Following this mechanism, studies have shown that catalase and/or other antioxidant enzymes in the cells contribute importantly to the cell’s sensitivity to pharmacological ascorbate, so as trans-metals that enhance ROS formation^13,24,39,40^. It is proposed that these mechanisms rendered cancer cells more susceptible than normal cells because cancer cells in these studies were found to have decreased ability to metabolize H_2_O_2_, or increased ability to form ROS. A recent study suggested that increased intracellular superoxide and H_2_O_2_ in cancer cells can disrupt ion metabolism and enhance intracellular redox cycling of ascorbate by iron ions contributing to selective toxicity and chemo-radio-sensitization^13^. However, sensitivity to pharmacological ascorbate treatment seems to involve multiple cellular components and functional pathways in addition to the capacity of metabolizing H_2_O_2_^14,20,41–43^. What is apparent is that peroxide formation, mediated by pharmacological ascorbate, is essential for cell death. A thorough understanding is yet to form on the cellular/molecular mechanisms of pharmacological ascorbate action. We postulate that Asc-induced ROS have multiple mechanisms of action on cells. Consistent with this hypothesis, many factors are suggested important in determining the fate of cancer cells after exposure to pharmacological ascorbate, such as hypoxia induced factor (HIF)^44^, KRAS/BRAF status^14^, P53 status^45^, DNA repair^42^, glucose and/or ascorbate transporters^46^. We showed in previous studies that Asc-generated ROS induces DNA damage and ATP depletion, causing downstream AMPK activation and mTOR inhibition^9^. Described here are several cellular responses that have not been elucidated before. Downstream to DNA damage induced by H_2_O_2_^9^, cellular NAD+ decreases as an effect of PARP activation^47^. Decrease of NAD+ triggers different effects in pancreatic cancer cells versus normal cells. First, ATP in pancreatic cancer cells decreases and leads to cell death, while normal cells maintain their ATP levels. This phenomenon has a root in dysregulated glucose metabolism in cancer cells, known as the Warburg Effect^33,48,49^, that renders cancer cells less efficient in ATP production than normal cells because they depend on a larger proportion on glycolysis for ATP whereas normal cells depend more on oxidative phosphorylation. Second, lack of NAD+ inhibits activity of Sirt-2, and induces tubulin acetylation, which in turn disrupts dynamics of microtubules. This influences cancer cells that are actively undergoing mitosis and migration. Further, pharmacological ascorbate inhibited EMT, an important process contributing to cancer metastasis. The mechanisms of Asc-induced EMT inhibition is worth further investigation. Finally, pharmacological ascorbate enhanced collagen synthesis in tumor stroma. Despite the controversial reports on the effect of elevated collagen in tumor progression^50–54^, the increased collagen by pharmacological ascorbate treatment is associated with restriction of tumor invasion in our animal experiment and in the patient description.

Targeting multiple pathways of cancer cells without affecting normal cells could decrease toxicities and the likelihood of emerging resistance, both being serious problems in cancer chemotherapies. Data here show multi-targeting effects of pharmacological ascorbate that favor death/inhibition in cancer cells relative to normal cells. Thus cell sensitivity to pharmacological ascorbate is not likely to be determined by any single pathway^43^. For a cancer that has complicated heterogeneity, such as pancreatic cancer, the multi-targeting actions of ascorbate are advantageous, because if inhibition of one target is incomplete, or ultimately fails because of mutations, treatment effects could be exerted through other pathways. A potential problem for multi-targeting agents is collateral/ multiple toxicities. However, pharmacological ascorbate is safe with few adverse events in animals and people^55^. Combination of this unique agent with current 1^st^ line chemotherapy or radiation therapy may result in additional synergistic effects in treating tumors.

The FDA recently approved the addition of nab-paclitaxel to gemcitabine (Nabpax + Gem) for treatment of advanced pancreatic cancer^5^. In a Phase III trial, Nabpax + Gem improved median overall survival (OS) to 8.5 month compared to 6.7 months with gemcitabine. One year survival was 35% and 2 year survival was 9%, significant increases compared to 22% and 4% with gemcitabine. A few smaller Phase II trials with 11–30 patients achieved 12–13.5 months median OS with Nabpax + Gem^56–59^. However, the Nabpax + Gem increased grade 3 or higher neutropenia, fatigue and neuropathy^5^. Our Phase I/IIa trial with data in 12 patients showed an encouraging median OS of 15.1 months by adding high dose intravenous ascorbate to gemcitabine (IVC + Gem). Survival at 1 year was 50%, and at 2 years was 8.3%. We realize that this trial cannot be compared to the Phase III trial using Nabpax + Gem. Our trial had a one-arm design, small sample size, a mixture of prior treated and untreated patients, and a mixture of locally advanced PDAC (4 participants, 33%) and metastatic PDAC (8 participants, 67%) at enrollment. Our results are in agreement with another published trial using similar treatment of IVC + Gem^12^, in which 9 subjects with biopsy-proven stage IV pancreatic cancer were treated with twice weekly IVC and co-current gemcitabine, aiming to establish tolerability and safety of IVC. The dose and duration of IVC treatment were similar in both the reported trial and our trial: escalating from 15 g to the aimed plasma ascorbate level of ~350 mg/dL (ranging from 50 to 125 g/infusion of ascorbate). Treatment duration was 69–556 days. Of the 9 patients completed the treatment, 6 maintained or improved performance status. The overall survival achieved 13 ± 2 months, similar to the 15.1 months in our trial. Notably, the patient in our trial who became resection eligible had metastatic disease on Stage III at enrollment, and had failed FOLFIRINOX.

We observed a mix of stable disease, partial response and disease progression in the course of our trial. Although RECIST criteria^60^ was used to assess tumor responses, overall response was not selected to be discussed when the trial was designed. With the focus on the Phase I pharmacokinetic analysis of ascorbate with gemcitabine and the intent to enroll based on the participants needed to determine that effect, it is not likely to be able to adequately evaluate response in these limited numbers. Also, our trial did not observe an improved progression-free survival (PFS), compared with gemcitabine or Nabpax + Gem^5^. Except for the small sample size, the lack of improvement in PFS could be secondary to the high drop-out rate and high percentage of pre-treated patients. Eight out of the twelve (67%) patients had failed prior treatments, and had progressive disease at the time of enrollment. Six patients (50%) had disease progression when on the trial. One patient had tumor response and was removed from the trial for surgery resection of the tumor, 4 dropped out because of unrelated medical issues and 1 dropped out for personal reason. These removed or dropped-out patients did not necessarily have disease progress while on the trial, but their withdrawals were counted as time to progress when calculating the PFS, causing an underestimation of PFS for this trial. A larger, more definitive Phase II/III trial is needed to detect efficacy and benefit of IVC.

Independent of the promises in treatment efficacy, an obvious advantage for using IVC in cancer treatment is its low-toxicity, which is now shown repeatedly in more than 10 recent clinical studies^61^. Here again, IVC did not add any significant adverse effects (SAE) to gemcitabine chemotherapy. IVC does not alter gemcitabine Cmax and AUC, only shortened gemcitabine half-life (T1/2) by 4 minutes, which was statistically significant. However, as gemcitabine T1/2 is only 16.7 minutes and is quickly metabolized to dFdU which has a much longer half-life of 12 hours, evaluation of dFdU half-life is more clinically meaningful. IVC does not change dFdU half-life, nor does it change Cmax or AUC of dFdU. The conclusion can be made that IVC does not influence gemcitabine pharmacokinetics in any clinically significant way. These data are the first to describe in detail that ascorbate does not change pharmacokinetic parameters of a standard chemotherapeutic agent.

## Methods

### Phase I/IIa Clinical Trial

A prospective phase I/IIa trial was conducted at the University of Kansas Medical Center (KUMC) Cancer Center and KU Integrative Medicine Clinic in Kansas City, Kansas. The KUMC Institutional Review Board approved the protocol and all participants were provided written informed consent. Oversight was provided by the US Food and Drug Administration’s Center for Drug Evaluation and Research, Division of Oncology Drug Products, with an Investigational New Drug assignment for injectable ascorbate. The trial was registered with http://www.ClinicalTrials.gov on May 24^th^, 2011 and assigned an identifier NCT01364805. All methods were performed in accordance with the relevant guidelines and regulations.

Independent data safety and monitoring oversight was provided by the KUMC Cancer Center Data Safety and Monitoring (DSMB) Committee. The primary objective was to assess safety combining high-dose IV ascorbate (IVC) with gemcitabine chemotherapy in the treatment of locally advanced or metastatic pancreatic cancer not eligible for surgical resection. The secondary objective was to determine if there was drug-drug interaction in terms of pharmacokinetics. Patients with newly diagnosed unresectable or metastatic pancreatic cancer who declined combination chemotherapy, or patients who progressed on a non-gemcitabine containing treatment were eligible for further screening, requiring them to be ambulatory with Eastern Cooperative Oncology Group (ECOG) performance status 0 to 2; have normal glucose-6-phosphate dehydrogenase (G6PD) status; have adequate renal, hepatic, and hematologic function; be able to receive chemotherapy for duration prescribed; and not use tobacco products.

Seven subjects were enrolled initially and when safety was confirmed an additional 7 participants were enrolled to total 14 participants. All participants received gemcitabine chemotherapy according to standard of care, and all doses were administered in the KUMC Cancer Center under the direction of the treating oncologist with oversight by co-investigator oncologist. Injectable ascorbic acid (Mylan, Inc. formerly Bioniche Pharma) dosing was established via dose escalation as described elsewhere^9^ (Table S3).

For Phase I, after dose escalation of IVC, pharmacokinetic evaluation of ascorbate alone was performed, followed by pharmacokinetic evaluation of gemcitabine and its metabolite 2-fluoro-2′-deoxyuridine (dFdU), and then IVC and gemcitabine were combined on the same day and pharmacokinetic evaluation was performed (Table S3). During this time, safety data was collected. Then subjects entered Phase IIa and received IVC 3× weekly in conjunction with established gemcitabine dose and schedule, until tumor progression determined by Response Evaluation Criteria in Solid Tumors (RECIST), or withdrawal for other reasons. All untoward events were evaluated, using the National Cancer Institute (NCI) Common Terminology Criteria for Adverse Events version 4.

Pharmacokinetic data were collected over a 24-hour period and were characterized in 12 subjects. Serial samples from blood draws were processed within 30 min of acquisition, and plasma were stored at −80 °C until analyzed. Doses of gemcitabine and ascorbate were 1000 mg/m^2^ and 100 g, respectively, with a few subjects received reduced doses as determined by the treating oncologist (Table S4). Compensation for the reduced doses was incorporated by using dose-normalization transformation for Cmax and AUC values.

### Orthotopic mouse model for pancreatic cancer

All procedures followed the animal protocol approved by the Institutional Animal Care and Use Committee at the University of Kansas Medical Center. PANC-1 cells were transfected with the lentivirus-expressing luciferase gene and stable expression cells were selected (PANC-1–Luc). Female Ncr nu/nu mice at 4–6 week of age were performed a small subcostal laparotomy while under anesthesia, with 2 × 10^5^ PANC-1-Luc cells injected into the tail of pancreas. Mice were imaged a week after cell implantation to monitor tumor formation. To image, each mouse was given 150 mg/kg D-luciferin by intraperitoneal injection. Animals were scanned using an IVIS imaging system (Waltham, MA). Mice were grouped to make the initial tumor burden even (n = 8 per group), and then were treated with intraperitoneal injection of ascorbate (Asc, 4 g/kg daily), or gemcitabine (Gem, 40 mg/kg every 3 days), or the combination of Asc and Gem. Control group was treated with saline with osmolarity equivalent to the ascorbate solution. Mice were imaged longitudinally. Treatment lasted for 45 days. At necropsy, total tumor burden were weighted, metastatic lesions in the abdomen were examine by gross necropsy. Tissue samples were fixed in formaldehyde, or spot-frozen on dry ice and stored at −80 °C for further analysis.

### Analytical methods

Concentrations of gemcitabine and 2-fluoro-2′-deoxyuridine (dFdU) were detected using a fully validated UPLC-MS/MS assay^38^. Concentrations of ascorbate were detected using an established method using HPLC coupled with electro-chemical detection^62^. Cmax, AUC and t ½ were calculated using Phoenix WinNonlin® v. 6.4 software.

### Cell culture and viability assay

hTERT-HPEN (immortalized human pancreatic ductal epithelial cells) was provided by Dr. Shrikant Anant at the University Of Kansas Cancer Center (Kansas City, KS). L3.6 was provided by Dr. Liang Xu at the University of Kansas (Lawrence, KS). Pan02 was donated by Dr. Anthony Sandler at Children’s National Medical Center (Washington DC). All other cell lines were obtained from the American Type Culture Collection (ATCC, Manassas, VA). All cells were cultured in recommended media supplemented with 10% fetal bovine serum (FBS), 100 units/ml penicillin/streptomycin at 37 °C in a humidified 5% CO2 atmosphere. Treatments were performed at ~70% confluency for all cells (approximately 0–0.4 nmoles/cell of ascorbate). MTT (4,5-dimethylthiazol-2-yl)-2,5-diphenyltetrazolium bromide) assay was used in determining cell viability. Formazan crystal was dissolved in DMSO and color measured at 570 nm.

Longer term cell survival was measured using colony formation assay in a 2-layer soft agar system, with top layer containing 0.5% and bottom layer containing 0.75% agar. Cells were seeded in 6-well plates at 1,000 to 2,500 cells per well (approximately 2–5 nmoles/cell of ascorbate). Ascorbate, catalase, or NAD+ were added at the time of seeding. Colonies were stained with crystal violet and counted after 21–28 days.

### Gene transfection

PANC-1 cells were engineered to overexpress a flag tagged HDAC6. Recombinant Plasmid pcDNA3.1+-HDAC6-flag and the empty vector pcDNA3.1+ were purchased from Addgene (Cambridge, MA). Plasmids were transfected into the PANC-1 cells with lipofectamine ^TM^ 2000 reagent (Invitrogen, Grand Island, NY) according to manufacturer instructions. G418 was used for selection of positive clones. Optimal dose of G418 was determined by MTT assay (IC_50_ = 0.625 mg/ml). Thirty-six hours after transfection, cells were passaged and were cultured in DMEM medium containing 10% FBS and 1 mg/ml G418. After 6 days, G418 was reduced to 0.5 mg/ml. G418 resistant clones were picked after two weeks and then expanded. Stable expression of HDAC6 in the clones was confirmed using western blot.

### RNA isolation, cDNA synthesis, and Real-Time PCR

Total RNA was extracted from cells by using TRIZOL reagent (Invitrogen, Grand Island, NY), or Nucleospin RNA (Macherey-Nagel, Deer Park, NY) according to the protocols of the manufacturers. cNDA synthesis was performed with 1 µg of total RNA using Omniscript RT kit according to manufacturer’s protocol (Quiagen, Valencia, CA), and was diluted 1:5 in autoclaved nanopure water for further analysis. Real-time PCR was performed using Bio-Rad iQ iCycler detection system with iQ SYBR green supermix (Bio-Rad Laboratories Ltd, Hercules, CA). Reactions were performed in a total volume of 10 µl, including 5 µl of 2X iQ SYBR green supermix, 1 µl of primers at 20 pmol/µl and 1 µl of cDNA template. All reactions were carried out in at least triplicates for every sample. Data were normalized to 18 S rRNA, or GAPDH.

### Matrigel invasion assay

Cells were seeded into inserts of Boyden chambers (BD Biosciences, San Jose, CA) that were either pre-coated or not coated with matrigel (0.1 mg/mL), at 1 × 10^4^ cells per insert in 0.5% FBS containing medium. Media in wells contained 10% FBS as chemo-attractor. After 24 h incubation, cells invaded to the bottom-side of the membrane were fixed with 4% para formaldehyde for 2 min, permeabilized with 100% methanol for 20 min, followed by staining with 0.05% crystal violet for 15 min at 37 °C. Non-invading cells on the top-side of the membrane were removed by cotton swab. Photographs were taken from five random fields per insert. Cells in the five random fields were counted.

### Gelatin zymography

Supernatant media from PANC-1 cell culture was subjected to electrophoresis on 10% SDS poly acryl amide gel containing 0.2% gelation (Sigma, St. Louis, MO). After adequate rinse in rinse buffer (1 M Tris pH8.0, 1 M CaCl2, 2.5% Triton X-100), the gel was equilibrated for 30 min in incubation buffer (1 M Tris pH8.0, 1 M CaCl2), and then incubated in fresh incubation buffer at 37 °C for 16 hrs. The gel was stained with Coomassie Brilliant Blue R-250 (Bio-Rad, Hercules, CA) for 1–2 h, and then destained in 10% methanol and 5% acetic acid. The clear bands corresponding to MMP activity were analyzed by optical densitometry by ImageJ software.

### Immunofluorescence and immunohistochemistry

Cells grown on 96 well plates were treated and then fixed in 4% paraformaldehyde, and blocked in blocking buffer (1X PBS+5% Goat serum+0.3%Triton X-100) at room temperature for 1hr. Anti-acetylated α-tubulin (Abcam, 1:4000 dilution in 1XPBS+1%BSA+0.3% triton X-100) was incubated at 4 °C for overnight. Alexa flour 488-conjugated secondary antibody (1:500) was incubated for 2 h in dark. Nuclei were visualized with 1 mg/mL Hoechst33342.

Paraffin-embedded tumor sections (5 µM thick) were deparaffinized and rehydrated by serial incubation in xylene, 100%, 95% ethanol, and water. Endogenous peroxide was blocked with 3% hydrogen peroxide at room temperature for 10 min. Antigen retrieval was performed in boiling citrate buffer for 5 min followed by sub boiling temperature for 10 min. Anti-PCNA primary antibody (1:1000, Cell Signaling Technology, Beverly, MA) was incubated overnight at 4 °C. Biotinylated secondary antibody and DAB were used to develop the blots (Vectastain ABC –AP kit, Vector Laboratories, Burlingame, CA). All the sections were counterstained with hematoxylin.

### Masson’s trichrome staining for collagen

Collagen content in the tumor and liver sections was detected by using Masson’s trichrome stain kit (Sigma, St. Louis, MO) following manufacturer’s protocol. The cytoplasm was stained a pink to red color and the collagen was stained blue.

### Native PAGE, SDS PAGE and western blot

Cells were lysed with RIPA buffer (25 mM Tris pH 7.6, 150 mM NaCl, 0.5% sodium deoxcholate, 1% NP-40, supplemented with 1 mM DTT and protease inhibitors), centrifuged, and supernatant was used. Protein quantification used BCA method (Pierce BCA protein assay kit, Waltham, MA). SDS-PAGE and Western blot was performed as routine. Native PAGE protocol was adapted from a recent publication^63^. Briefly, 10 µg protein was mixed with equal volume of 2 × native sample buffer (Bio-Rad laboratories, Ltd, Hercules, CA), and were loaded onto 8% poly acryl amide gels without SDS. The electrophoresis was at 60 V for 3.5–4 hour. Proteins were transferred to PVDF membrane for overnight at 4 °C. Dilutions for primary antibodies were anti-α-tubulin, anti-HDAC6 anti-vinculin, (1:1000, Cell Signaling Technology, Beverly, MA), anti-acetylated α-tubulin (1:5000), anti-Sirt-2 (1:500), anti-α-TAT (1:1000), anti-flag (1:1000) (Sigma Aldrich, St. Louis, MO). A goat anti-rabbit and anti-mouse polyclonal horseradish peroxidase (HRP) conjugated secondary antibody (1:1000, Cell Signaling Technology, Beverly, MA) was used. Blots were established using a chemiluminescence detection kit (Pierce ECL western blotting substrate, Thermo Scientific, Rockford, IL).

### Statistics

Statistical analysis was performed using SYSTAT 11 software for student T-test for comparison between 2 groups, and using ANOVA with post-hoc Bonferroni and Holm analysis when comparison involves more than 2 groups, with all groups simultaneously compared. A difference was considered significant at the p < 0.05 level. Correlation analysis used the standard Pears Tests. Differences between individual pharmacokinetic parameters for gemcitabine, dFdU, and ascorbate determined from the single agent treatment and from the combined treatment were assessed using a paired t-test when data sets met the Shapiro-Wilk test of normality, or using the Wilcoxon signed rank test when normality was not observed.

### Study Approval

The clinical trial was approved by the KUMC Institutional Review Board. All participants were provided written informed consent at enrollment. Oversight was provided by the US Food and Drug Administration’s Center for Drug Evaluation and Research, Division of Oncology Drug Products, with an Investigational New Drug assignment for injectable ascorbic acid. The trial was registered with http://www.ClinicalTrials.gov and assigned an identifier NCT01364805. Independent data safety and monitoring oversight was provided by the KUMC Cancer Center Data Safety and Monitoring (DSMB) Committee.

The animal protocol was approved by the Institutional Animal Care and Use Committee at the University of Kansas Medical Center.

## Electronic supplementary material

Supplementary figures and tables

## References

[1] American Cancer Society. Cancer Facts and Figures 2017 (2017).

[2] Burris H (1997). Improvements in survival and clinical benefit with gemcitabine as first-line therapy for patients with advanced pancreas cancer: a randomized trial. Journal of Clinical Oncology.

[3] Renouf D, Moore M (2010). Evolution of systemic therapy for advanced pancreatic cancer. Expert Rev Anticancer Ther.

[4] Kim R (2011). FOLFIRINOX: a new standard treatment for advanced pancreatic cancer?. Lancet Oncol.

[5] Von Hoff DD (2013). Increased survival in pancreatic cancer with nab-paclitaxel plus gemcitabine. N Engl J Med.

[6] Moore MJ (2007). Erlotinib plus gemcitabine compared with gemcitabine alone in patients with advanced pancreatic cancer: a phase III trial of the National Cancer Institute of Canada Clinical Trials Group. J Clin Oncol.

[7] Dahan L (2010). Combination 5-fluorouracil, folinic acid and cisplatin (LV5FU2-CDDP) followed by gemcitabine or the reverse sequence in metastatic pancreatic cancer: final results of a randomised strategic phase III trial (FFCD 0301). Gut.

[8] Oberstein PE, Saif MW (2011). First-line treatment for advanced pancreatic cancer. Highlights from the “2011 ASCO Gastrointestinal Cancers Symposium”. San Francisco, CA, USA. January 20–22, 2011. JOP.

[9] Ma Y (2014). High-dose parenteral ascorbate enhanced chemosensitivity of ovarian cancer and reduced toxicity of chemotherapy. Science translational medicine..

[10] Hoffer LJ (2008). Phase I clinical trial of i.v. ascorbic acid in advanced malignancy. Ann Oncol.

[11] Monti DA (2012). Phase I evaluation of intravenous ascorbic acid in combination with gemcitabine and erlotinib in patients with metastatic pancreatic cancer. PLoS One.

[12] Welsh JL (2013). Pharmacological ascorbate with gemcitabine for the control of metastatic and node-positive pancreatic cancer (PACMAN): results from a phase I clinical trial. Cancer Chemother Pharmacol.

[13] Schoenfeld, J. D. *et al*. O2- and H2O2-Mediated Disruption of Fe Metabolism Causes the Differential Susceptibility of NSCLC and GBM Cancer Cells to Pharmacological Ascorbate. *Cancer Cell*, 10.1016/j.ccell.2017.02.018 (2017). 10.1016/j.ccell.2017.07.008 28810149

[14] Yun J (2015). Vitamin C selectively kills KRAS and BRAF mutant colorectal cancer cells by targeting GAPDH. Science.

[15] Jones S (2008). Core signaling pathways in human pancreatic cancers revealed by global genomic analyses. Science.

[16] Graumlich JF (1997). Pharmacokinetic model of ascorbic acid in healthy male volunteers during depletion and repletion. Pharm Res.

[17] Levine M (1996). Vitamin C pharmacokinetics in healthy volunteers: evidence for a recommended dietary allowance. Proc Natl Acad Sci USA.

[18] Padayatty SJ (2004). Vitamin C pharmacokinetics: implications for oral and intravenous use. Ann Intern Med.

[19] Chen Q (2008). Pharmacologic doses of ascorbate act as a prooxidant and decrease growth of aggressive tumor xenografts in mice. Proc Natl Acad Sci USA.

[20] Chen Q (2007). Ascorbate in pharmacologic concentrations selectively generates ascorbate radical and hydrogen peroxide in extracellular fluid *in vivo*. Proc Natl Acad Sci USA.

[21] Chen Q (2005). Pharmacologic ascorbic acid concentrations selectively kill cancer cells: action as a pro-drug to deliver hydrogen peroxide to tissues. Proc Natl Acad Sci USA.

[22] Parrow NL, Leshin JA, Levine M (2013). Parenteral ascorbate as a cancer therapeutic: a reassessment based on pharmacokinetics. Antioxid Redox Signal.

[23] Du J (2010). Mechanisms of ascorbate-induced cytotoxicity in pancreatic cancer. Clin Cancer Res.

[24] Verrax J, Calderon PB (2009). Pharmacologic concentrations of ascorbate are achieved by parenteral administration and exhibit antitumoral effects. Free Radic Biol Med.

[25] Pollard HB, Levine MA, Eidelman O, Pollard M (2010). Pharmacological ascorbic acid suppresses syngeneic tumor growth and metastases in hormone-refractory prostate cancer. In Vivo.

[26] Takemura Y (2010). High dose of ascorbic acid induces cell death in mesothelioma cells. Biochem Biophys Res Commun.

[27] Deubzer B (2010). H(2)O(2)-mediated cytotoxicity of pharmacologic ascorbate concentrations to neuroblastoma cells: potential role of lactate and ferritin. Cell Physiol Biochem.

[28] Espey MG (2011). Pharmacologic ascorbate synergizes with gemcitabine in preclinical models of pancreatic cancer. Free Radic Biol Med.

[29] Rhim AD (2012). EMT and dissemination precede pancreatic tumor formation. Cell.

[30] Ellenrieder V (2000). Role of MT-MMPs and MMP-2 in pancreatic cancer progression. Int J Cancer.

[31] Chiarugi A, Moskowitz MA (2002). Cell biology. PARP-1–a perpetrator of apoptotic cell death? Science.

[32] Pero RW (1990). Oxidative stress induces DNA damage and inhibits the repair of DNA lesions induced by N-acetoxy-2-acetylaminofluorene in human peripheral mononuclear leukocytes. Cancer Res.

[33] Warburg O, Wind F, Negelein E (1927). The Metabolism of Tumors in the Body. J Gen Physiol.

[34] Sauve AA, Youn DY (2012). Sirtuins: NAD(+)-dependent deacetylase mechanism and regulation. Current opinion in chemical biology.

[35] Perdiz D, Mackeh R, Pous C, Baillet A (2011). The ins and outs of tubulin acetylation: more than just a post-translational modification?. Cellular signalling.

[36] Hammond JW, Cai D, Verhey KJ (2008). Tubulin modifications and their cellular functions. Current opinion in cell biology.

[37] Kirkland WL, Burton PR (1972). Cyclic adenosine monophosphate-mediated stabilization of mouse neuroblastoma cell neuritis microtubules exposed to low temperature. Nat New Biol.

[38] Marangon E (2008). Simultaneous determination of gemcitabine and its main metabolite, dFdU, in plasma of patients with advanced non-small-cell lung cancer by high-performance liquid chromatography-tandem mass spectrometry. J Mass Spectrom.

[39] Doskey CM (2016). Tumor cells have decreased ability to metabolize H2O2: Implications for pharmacological ascorbate in cancer therapy. Redox biology.

[40] Du J, Wagner BA, Buettner GR, Cullen JJ (2015). Role of labile iron in the toxicity of pharmacological ascorbate. Free Radic Biol Med.

[41] Venturelli S (2014). Epigenetic impacts of ascorbate on human metastatic melanoma cells. Frontiers in oncology.

[42] Castro ML, McConnell MJ, Herst PM (2014). Radiosensitisation by pharmacological ascorbate in glioblastoma multiforme cells, human glial cells, and HUVECs depends on their antioxidant and DNA repair capabilities and is not cancer specific. Free Radic Biol Med.

[43] Levine M, Violet PC (2017). Data Triumph at C. Cancer Cell.

[44] Sinnberg T (2014). The ROS-induced cytotoxicity of ascorbate is attenuated by hypoxia and HIF-1alpha in the NCI60 cancer cell lines. J Cell Mol Med.

[45] Kim J (2012). Enhanced antitumor activity of vitamin C via p53 in cancer cells. Free Radic Biol Med.

[46] Hong SW (2013). SVCT-2 in breast cancer acts as an indicator for L-ascorbate treatment. Oncogene.

[47] Ma E (2017). Pharmacologic ascorbate induces neuroblastoma cell death by hydrogen peroxide mediated DNA damage and reduction in cancer cell glycolysis. Free Radic Biol Med.

[48] Xu RH (2005). Inhibition of glycolysis in cancer cells: a novel strategy to overcome drug resistance associated with mitochondrial respiratory defect and hypoxia. Cancer Res.

[49] Ahmad IM (2005). Mitochondrial O2*- and H2O2 mediate glucose deprivation-induced stress in human cancer cells. J Biol Chem.

[50] Karnoub AE (2007). Mesenchymal stem cells within tumour stroma promote breast cancer metastasis. Nature.

[51] Merika EE, Syrigos KN, Saif MW (2012). Desmoplasia in pancreatic cancer. Can we fight it?. Gastroenterol Res Pract.

[52] Bissell MJ, Radisky D (2001). Putting tumours in context. Nat Rev Cancer.

[53] Dvorak HF (1986). Tumors: wounds that do not heal. Similarities between tumor stroma generation and wound healing. N Engl J Med.

[54] Ozdemir BC (2014). Depletion of carcinoma-associated fibroblasts and fibrosis induces immunosuppression and accelerates pancreas cancer with reduced survival. Cancer Cell.

[55] Chen Q, Polireddy K, Chen P, Dong R (2015). The unpaved journey of vitamin C in cancer treatment. Can J Physiol Pharmacol.

[56] Zhang DS (2013). Phase I/II study of albumin-bound nab-paclitaxel plus gemcitabine administered to Chinese patients with advanced pancreatic cancer. Cancer Chemother Pharmacol.

[57] Goldstein, D. *et al*. nab-Paclitaxel plus gemcitabine for metastatic pancreatic cancer: long-term survival from a phase III trial. *J Natl Cancer Inst* **107**, 10.1093/jnci/dju413 (2015). 10.1093/jnci/dju413 25638248

[58] Chiorean EG (2016). CA19-9 decrease at 8 weeks as a predictor of overall survival in a randomized phase III trial (MPACT) of weekly nab-paclitaxel plus gemcitabine versus gemcitabine alone in patients with metastatic pancreatic cancer. Ann Oncol.

[59] Ueno H (2016). Phase I/II study of nab-paclitaxel plus gemcitabine for chemotherapy-naive Japanese patients with metastatic pancreatic cancer. Cancer Chemother Pharmacol.

[60] Eisenhauer E (2009). New response evaluation criteria in solid tumors: revised RECIST guideline (version 1.1). Eur J Cancer.

[61] Chen Q, Polireddy K, Chen P, Dong R (2015). The unpaved journey of vitamin C in cancer treatment. Can J Physiol Pharmacol.

[62] Ma Y (2013). A Convenient Method for Measuring Blood Ascorbate Concentrations in Patients Receiving High-Dose Intravenous Ascorbate. Journal of the American College of Nutrition.

[63] Bollu LR (2014). Involvement of de novo synthesized palmitate and mitochondrial EGFR in EGF induced mitochondrial fusion of cancer cells. Cell cycle.

