# Supplementary material for: High Dose Parenteral Ascorbate Inhibited Pancreatic Cancer Growth and Metastasis: Mechanisms and a Phase I/IIa study
Source: Sci Rep. 2017 Dec 7;7:17188. doi: 10.1038/s41598-017-17568-8 (PMC5719364; doi:10.1038/s41598-017-17568-8)
Supplement: Supplementary file 1 — Supplementary figures and tables [file 41598_2017_17568_MOESM1_ESM.docx]

**High Dose Parenteral Ascorbate Inhibited Pancreatic Cancer Growth and Metastasis: Mechanisms and a Phase I/IIa study**

**Authors:** Kishore Polireddy^1,2^, Ruochen Dong^1,2^, Gregory Reed^1^, Jun Yu^1,2^, Ping Chen^1,2^, Stephen Williamson^3^, Pierre-Christian Violet^5^, Ziyan Pessetto^4^, Andrew K Godwin^4^, Fang Fan^4^, Mark Levine^5^, Jeanne A Drisko^2,3, *^, Qi Chen^1,2,*^

**Affiliations:**

^1^ Department of Pharmacology, Toxicology and Therapeutics; ^2^ KU Integrative Medicine; ^3^ Department of Internal Medicine, Hematology and Oncology Division; ^4^ Department of Pathology and Laboratory Medicine, University of Kansas Medical Center, Kansas City, KS, 66160. ^5^National Institute of Diabetes, Digestive and Kidney Diseases, the National Institutes of Health, Bethesda, MD 20892.

Supplementary Data:

**Fig. S1**

**
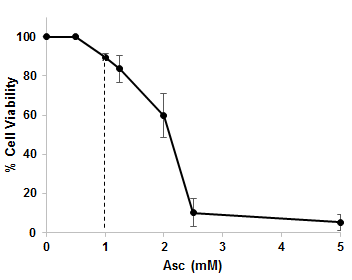
**

**Figure S1. Dose-response curve of ascorbate inhibiting PANC-1 cell viability.** PANC-1 cells in log-growth phase were incubated with indicated concentrations of ascorbate for 48 hrs. Cell viability was detected by MTT assay. Data represent average ± SD of 3 independent experiments each done in triplicates.

Fig. S2


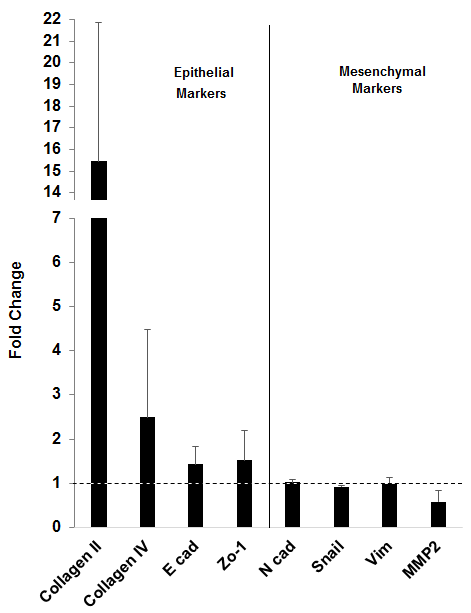


**Figure S2.** qRT-PCR for changes in EMT markers in MIA PaCa2 cells. Cells were treated with 1.25 mM Asc for 6 h. Data was normalized to 18s rRNA, and then compared to untreated MIA PaCa2 cells for fold change. Data represent Mean ± SD of 2-3 independent experiments.

**Fig. S2**

**A**

**B**

**
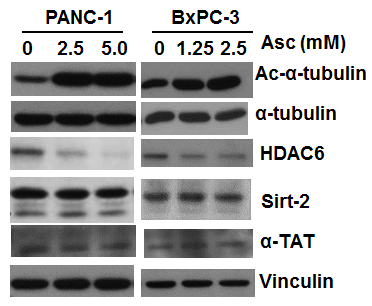

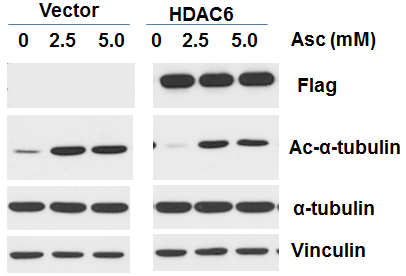
**

**C**

**Figure S2. Western blots of Sirt-2, HDAC6 and α-TAT after ascorbate treatment.** (**A**) Western blot analysis for Sirt-2, HDAC6 and α-TAT. PANC-1 and BxPC-3 cells were treated with ascorbate for 4 h. (**B**) Overexpression of Flag tagged HDAC6 in PANC-1 cells and α-tubulin acetylation induced by ascorbate (4 h). Vinculin was a loading control. (C) Sensitivity of PANC-1 cells and PANC-1 cells overexpressing HDAC6 to ascorbate treatment. Stable overexpression clone (#6) was selected using G418 after transfection with pDDNA3.1-HDAC6. Cells were treated for 48 h and viability was detected by MTT assay. Data represent Mean ± SD of 2 independent experiments each done in triplicates.

**Fig. S3**

**A**


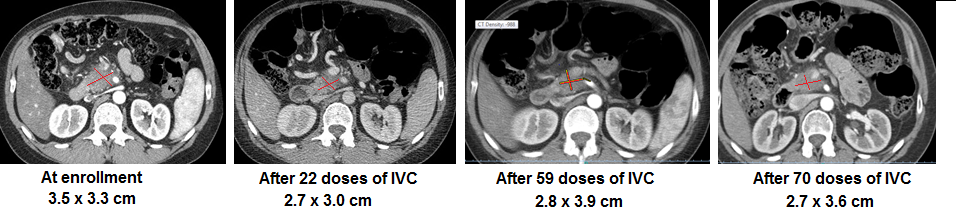


**B**


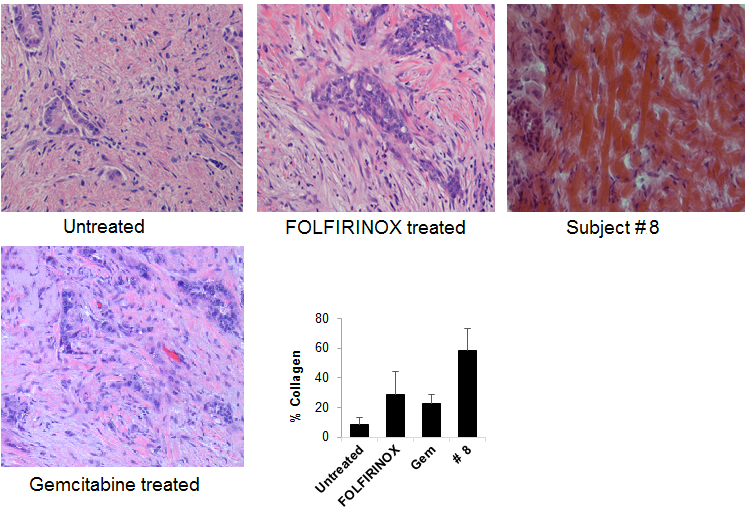


**Figure S3. Tumor Response of subject #8 to the combination treatment of IVC and gemcitabine. A**. PET-CT images. Subject #8 was diagnosed in 10/2013 with Stage III pancreatic ductal carcinoma not eligible for surgical resection. Initially the subject was treated with FOLFIRINOX for 6 months and disease progressed. At enrollment (5/13/2014), imaging showed infiltrating pancreatic mass of 3.5 cm × 3.3 cm. The second image on 7/18/2014 was taken when the subject had 22 doses of IVC at 100 g/infusion and 3 cycles of gemcitabine at the dose of 1,000 gm/m^2^. There was interval improvement in appearance of mass margins, which are less indiscreet. The third image was on 10/28/2014 when patient received 59 doses of IVC and 8 cycles of gemcitabine. The last image was on 1/21/2015 after patient had 70 doses of IVC and 9 cycles of gemcitabine. Patient was considered eligible for surgery removal of the tumor at this point. **B**. H&E staining of the resected tumor, in comparison to an untreated pancreatic cancer, a FOLFIRINOX treated pancreatic cancer, and a gemcitabine treated pancreatic cancer all from archived pathological slides. Collagen was stained pink/red. Bar graph represents Mean ± SD of % area collagen/cross section, 24 fields from 6 different slices were analyzed for subject #8, and 5 different fields were analyzed for each of the other samples.

**Table S1**

| **Cell line** | ***K-Ras*** | ***P53*** | ***CDKN2A/p16*** | ***SMAD4/DPC4*** |
| --- | --- | --- | --- | --- |
| BxPC-3 | WT | 220 Cys | WT | HD |
| AsPC-1 | 12 Asp | 135 Δ1 bp | WT | WT |
| MIA PaCa2 | 12 Cys | 248 Trp | HD | WT |
| Su86.86 | 12 Asp | 245 Ser | HD | WT |
| HPAFII | 12 Asp | 151 Ser | Δ20–25 Δ26–27 Δ29–34 | WT |
| HS 766T | 61 His | Mut 225–282  Δexons 2–4 | Intron 2 splice site | HD |
| PANC-1 | 12 Asp | 273 His, 273 Cys | HD | WT |
| L3.6pl | 12 Asp |  |  | Δ |

**Table S1 Genotype of the human pancreatic cancer cell lines used, showing the most common genetic alternations in pancreatic cancer** (63, 64). WT—wild type, Δ—deletion, bp—base pair, HD—homozygous deletion, Mut225-282— mutations found between codons 225-282

**Table S2a Demographics of Study Population**

| ID | Age | Gender | Prior Therapy before Enrollment | | Stage Initial Diagnosis | Stage Enrollment | ECOG at Enrollment |
| --- | --- | --- | --- | --- | --- | --- | --- |
| 1 | 76 | F | None | IV (T2, N1, M1) | | IV (T2, N1, M1) | 1 |
| 2 | 69 | F | Gemcitabine and Kanglaite X 2 months until progression | IV (T3, N1, M1) | | IV (T3, N1, M1) | 1 |
| 3 | 56 | M | FOLFIRINOX X 3 months until progression | IV (T3, N1, M1) | | IV (T3, N1, M1) | 1 |
| 4 | 76 | M | Gemcitabine X 1 month until enrollment in IV C trial | II (T2, N1, M0) | | IV (T2, N1, M1) | 2 |
| 5 | 52 | F | Capecitabine and Radiation X 5 months until progression | IIA (T3, N0, M0) | | III (T4, N0, M0) | 1 |
| 6 | 77 | F | None | III (T2, N0, M1) | | IV (T2, N0, M1) | 2 |
| 7 | 80 | F | None | IIA (T3, N0, M0) | | IIA (T3, N0, M0) | 0 |
| 8 | 41 | F | FOLFIRINOX X 6 months until progression | III (T2, N2, M0) | | III (T2, N2, M0) | 0 |
| 9 | 73 | F | Gemcitabine X 4 months | IV (T2, N1, M1) | | IV (T2, N1, M1) | 1 |
| 10 | 74 | M | None | III (T2, N1, M0) | | III (T2, N1, M0) | 1 |
| 11 | 36 | F | Capecitabin & radiation followed by surgery; FOLFIRINOX, gemcitabine X 19 months until progression | IIA (T3, N0, M0) | | IV (T2, N1, M1) | 1 |
| 12 | 53 | F | Gemcitabine and FOLFIRINOX X 18 months until progression | IV (T3, N1, M1) | | IV (T3, N1, M1) | 1 |
| 13 | 67 | M | FOLFIRINOX, FOLFIRI, surgical resection X 20 months until progression | I (T2, N0, M0) | | IV | 1 |
| 14 | 60 | F | FOLFIRINOX, FOLFIRI X 9 months until progression | IV | | IV | 1 |

**Table S2b Study Population Treatment Related Information**

| ID | IVC Dose (g) | Treatment Duration | Phase II # of IVC Infusions | Phase II Gemcitabine Treatment | Endpoint |
| --- | --- | --- | --- | --- | --- |
| 1 | 75 | 9 weeks | 26 | 4 cycles | Disease progression |
| 2 | 100 | 17 weeks | 48 | 6 cycles | Disease progression |
| 3 | 100 | 7 weeks | 26 | 3 cycles | Disease progression |
| 4 | 75 | 6 weeks | 16 | 3 cycles | Disease progression |
| 5 | 100 | 32 weeks | 92 | 9 cycles | Disease progression |
| 6 | 100 | 18 weeks | 51 | 6 cycles | Disease progression |
| 7 | 100 | 44 weeks | 158 | 13 cycles | Withdrew |
| 8 | 100 | 25 weeks | 70 | 9 cycles | Withdrew after imaging revealed response – tumor shrinkage and became eligible for surgical resection |
| 9 | 100 | 8 weeks | 18 | 2 cycles | Withdrew secondary to hip fracture and inability to continue with protocol requirements |
| 10 | 100 | 4 weeks | 9 | 1 cycle | Death unrelated to study drugs |
| 11 | 100 | 3 weeks | 3 | 1 cycle | Death from opioid overdoses at home |
| 12 | 100 | 1 week | 1 | 0 cycles | Withdrawal for personal reasons |
| 13 | 100 | 0 | 0 | 0 | Withdrew because of cerebrovascular accident |
| 14 | 100 | 0 | 0 | 0 | Withdrew for personal reasons |

**Table S3 Time schedule of Phase I pharmacokinetic study**

|  | **Week 1** | **Week 2** | | **Week 3** | **Week 4** |
| --- | --- | --- | --- | --- | --- |
| **Gem** | None | None | | PK | PK for Gem+IVC |
| **IVC** | Dose escalation: 25g, 50g and 75g | Dose escalation: 100 g | PK on final dose | None |  |

Gem, gemcitabine; IVC, intravenous ascorbate; PK, pharmacokinetics

**Table S4 Individual patient dosing**

| **Patient** | **Gemcitabine dose as single drug (mg/m2)** | **Gemcitabine in combination (mg/m2)** | **Ascorbate dose as single drug (g)** | **Ascorbate dose in combination (g)** |
| --- | --- | --- | --- | --- |
| 1 | 1000 | 1000 | 75 | 75 |
| 2 | 1000 | 1000 | 100 | 100 |
| 3 | 1000 | 1000 | 100 | 100 |
| 4 | 1000 | 1000 | 75 | 75 |
| 5 | 1000 | 1000 | 100 | 100 |
| 6 | 1000 | 1000 | 100 | 100 |
| 7 | 1000 | 750 | 100 | 100 |
| 8 | 1000 | 715 | 100 | 100 |
| 9 | 1000 | 1000 | 100 | 100 |
| 10 | 1000 | 750 | 100 | 100 |
| 11 | 800 | 800 | 100 | 100 |
| 12 | 1000 | 1000 | 100 | 100 |

**Table S5 Gemcitabine and dFdU pharmacokinetic parameters when gemcitabine was administered as single drug or in combination with IVC**

|  |  | **T ½,**  **h** | **Cmax,**  **µg/mL** | **Cmax/D’**  **(µg/mL)/gm** | **AUC,**  **h* µg/mL** | **AUC/D,**  **(h* µg/mL)/gm** |
| --- | --- | --- | --- | --- | --- | --- |
| Gem | Single-drug | 0.28 ± 0.04  (0.21 – 0.34) | 16.9 ± 5.6  (8.9 – 24.5) | 0.017 ± 0.005  (0.009 – 0.024) | 10.4 ± 3.3  (6.0 – 16.3) | 0.011 ± 0.003  (0.006 – 0.016) |
|  | combination | 0.25 ± 0.03  (0.20 – 0.30) | 14.2 ± 4.0  (7.1 – 21.0) | 0.016 ± 0.004  (0.009 – 0.023) | 9.2 ± 2.6  (4.5 – 13.1) | 0.010 ± 0.003  (0.006 – 0.018) |
|  |  | P =0.003 | P = 0.187 | P = 0.215 | P = 0.327 | P = 0.449 |
| dFdU | Single-drug | 12.2 ± 2.6  (8.1 – 16.7) | 32.9 ± 5.7  (23.9 – 42.0) | 0.033 ± 0.005  (0.026 – 0.042) | 258. ± 73.  (162. – 425.) | 0.262 ± 0.069  (0.174 – 0.425) |
|  | combination | 12.1 ± 2.2  (7.9 – 15.4) | 31.0 ± 4.8  (21.7 – 38.5) | 0.034 ± 0.003  (0.028 – 0.039) | 240. ± 60.  (155. – 377.) | 0.262 ± 0.056  (0.190 – 0.377) |
|  |  | P = 0.785 | P = 0.380 | P = 0.779 | P = 0.506 | P = 0.997 |
